# Supplementary figures and images for: Genetic variation of BnaA3.NIP5;1 expressing in the lateral root cap contributes to boron deficiency tolerance in Brassica napus
Source: PLoS Genet. 2021 Jul 1;17(7):e1009661. doi: 10.1371/journal.pgen.1009661 (PMC8279314; doi:10.1371/journal.pgen.1009661)

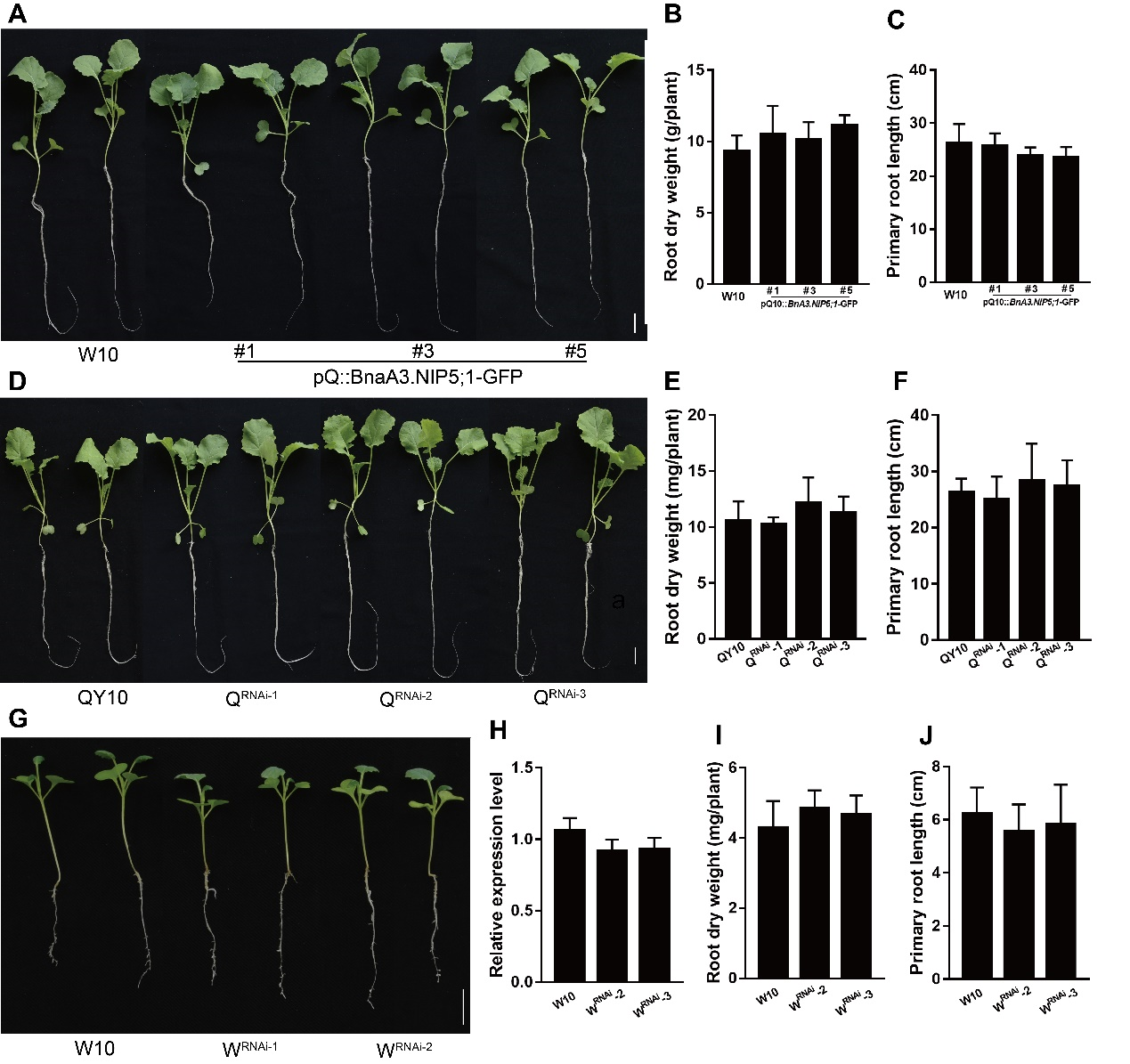

Supplement: S1 Fig — (A) Phenotype of 15-d-old wild-type (W10) and pQ::BnaA3.NIP5;1-GFP transgenic plants grown under normal B (100 μM) condition. Scale bar, 2 cm. (B and C) Root dry weight and primary root length of W10 and pQ::BnaA3.NIP5;1-GFP transgenic plants under normal B (100 μM) condition. Data presented are mean values with s.d. (n = 6). (D) Seedlings of 15-d-old wild-type (QY10) and QRNAi transgenic plants grown under normal B (100 μM) condition. Scale bar, 2 cm. (E and F) Root dry weight and primary root length of QY10 and QRNAi transgenic plants under normal B (100 μM) condition. Data presented are mean values with s.d. (n = 6). (G) Phenotype of 15-d-old wild-type (W10) and W10RNAi lines grown under low B (0.25 μM) condition. Scale bar, 2 cm. (H-J) Relative expression, root dry weight and primary root length of W10 and W10RNAi lines under low B (0.25 μM) condition. Data presented are mean values with s.d. (n = 3 in (H) and n = 6 in (I and J)). (TIF) [file pgen.1009661.s001.tif]

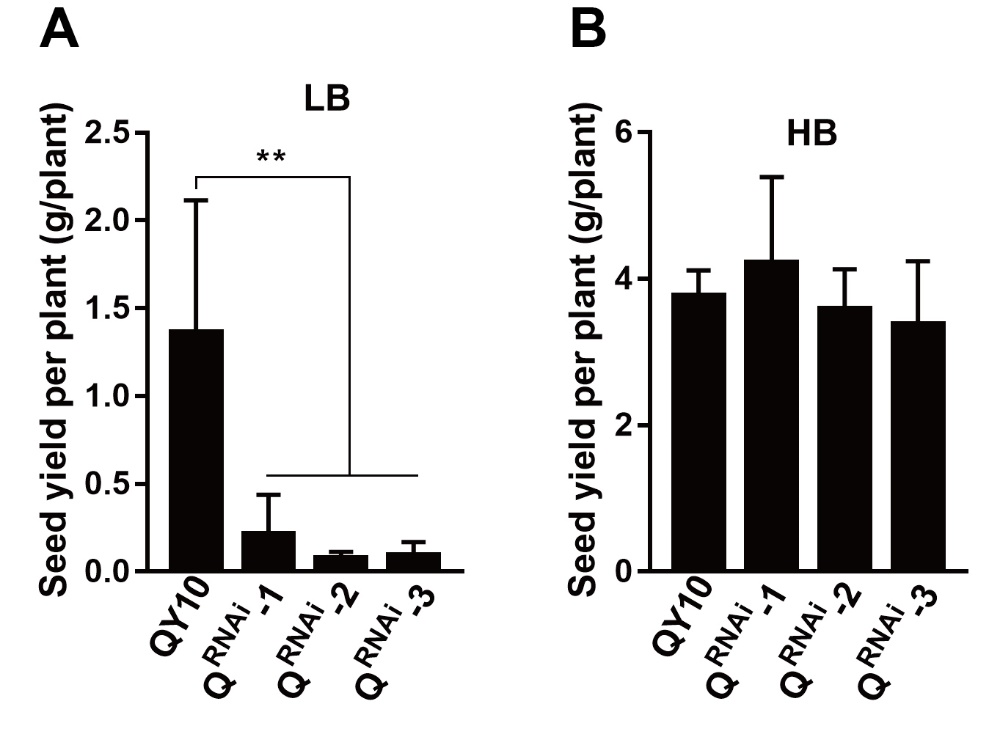

Supplement: S2 Fig — (A and B) Seed yield of wild-type (QY10) and QRNAi transgenic plants under low-B (LB, 0.25 mg kg-1) (A) and high-B (HB, 1.0 mg kg-1) (B) conditions by pot culture. Data presented are mean values of four replicates with s.d. ** P<0.01 (Student’s t-test). (TIF) [file pgen.1009661.s002.tif]

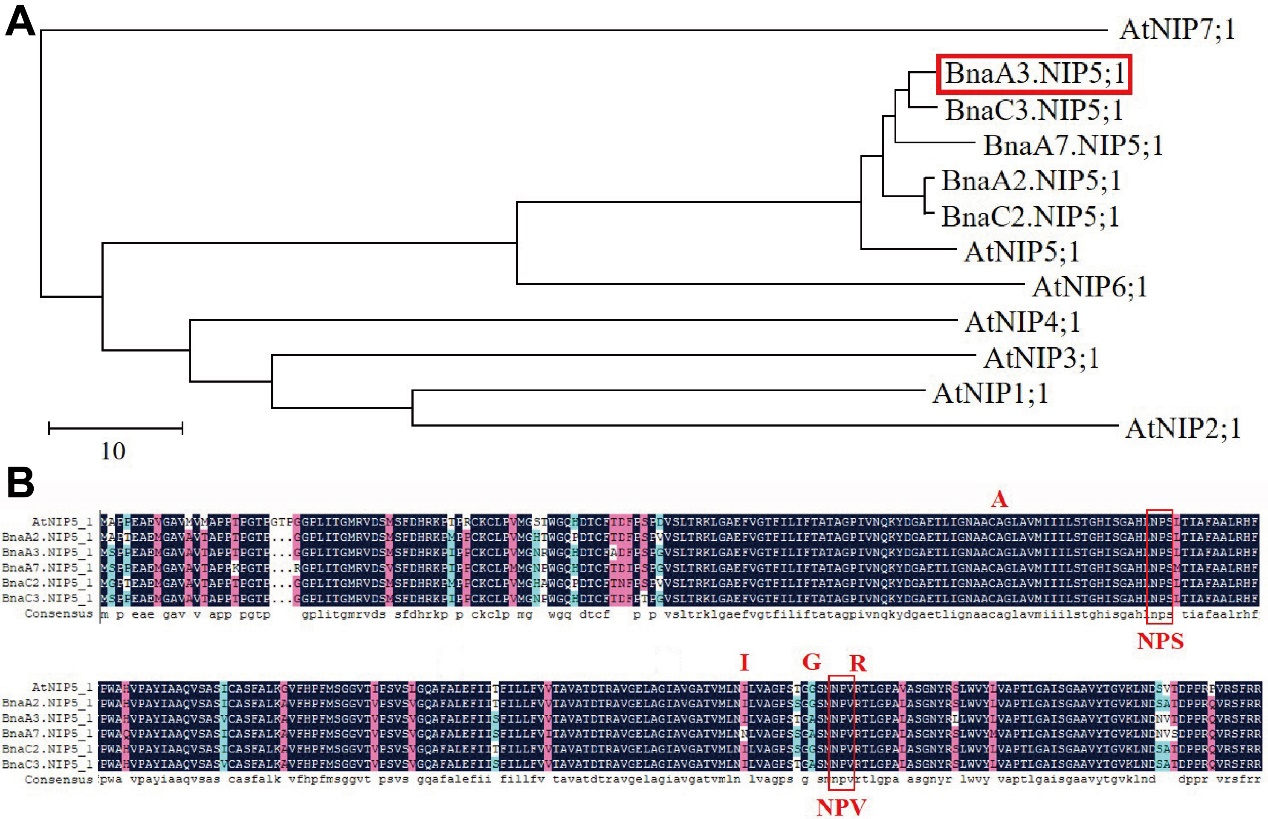

Supplement: S3 Fig — (A) Phylogenetic analysis of NIP proteins in Arabidopsis and B. napus. The phylogenetic tree was constructed using MEGA 5.10 software by Neighbor-Jointing method with 1000 bootstrapping trials. (B) Alignment of BnaNIP5;1s with AtNIP5;1. NPA motifs and ar/R are highlighted in red. (TIF) [file pgen.1009661.s003.tif]

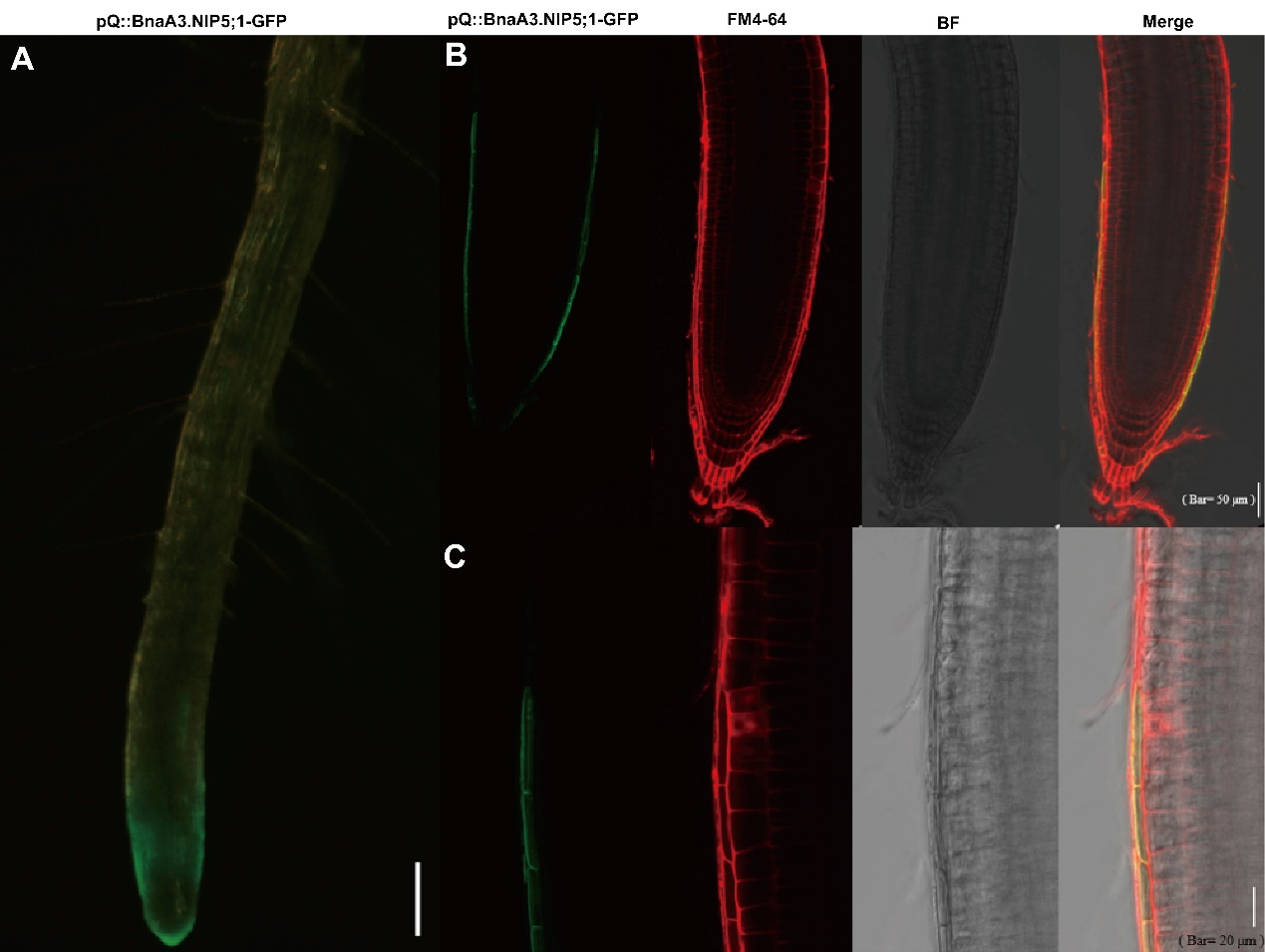

Supplement: S4 Fig — (A) Image of pQ::BnaA3.NIP5;1-GFP in Arabidopsis root performed by fluorescence microscopy (OLYMPUS SZX 16). Scar bar, 100 μm. (B and C) Confocal images of pQ::BnaA3.NIP5;1-GFP in Arabidopsis root performed by confocal microscopy (TCS SP8, Leica), FM4-64 was used as an membrane-selective tracer. Scar bar, 50 μm in (B) and 20 μm in (C). (TIF) [file pgen.1009661.s004.tif]

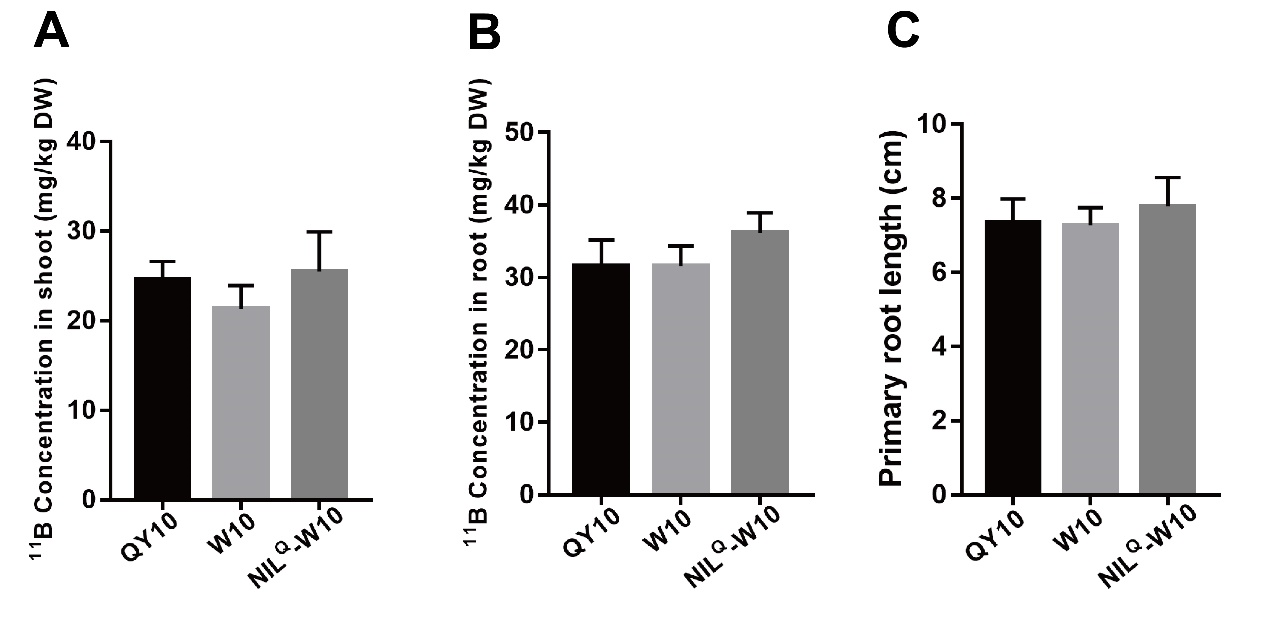

Supplement: S5 Fig — (A and B) 11B concentration in shoot (A) and root (B) in QY10, W10 and NILQ-W10. Seedlings of QY10, W10 and NILQ-W10 were pre-cultured with 25 μM 11B for 15 d, and then exposed to a solution containing 0 B for 1 d. Subsequently, the plants were exposed to 10 μM 10B for 1 h. Shoot and root were sampled separately and subjected to 10B and 11B determination by ICP-MS. Data presented are mean values of with s.d. (n = 3). (C) Primary root length of QY10, W10 and NILQ-W10 grown on 100 μM B solid medium for 5 d. Data presented are mean values of with s.d. (n = 16). (TIF) [file pgen.1009661.s005.tif]

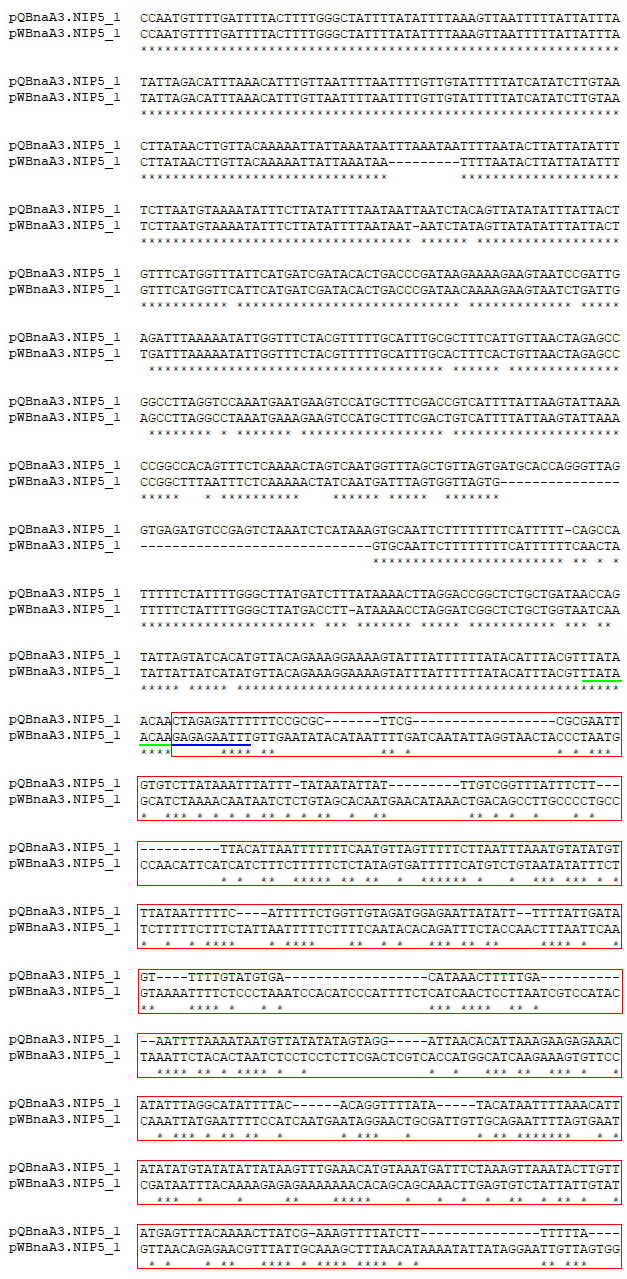


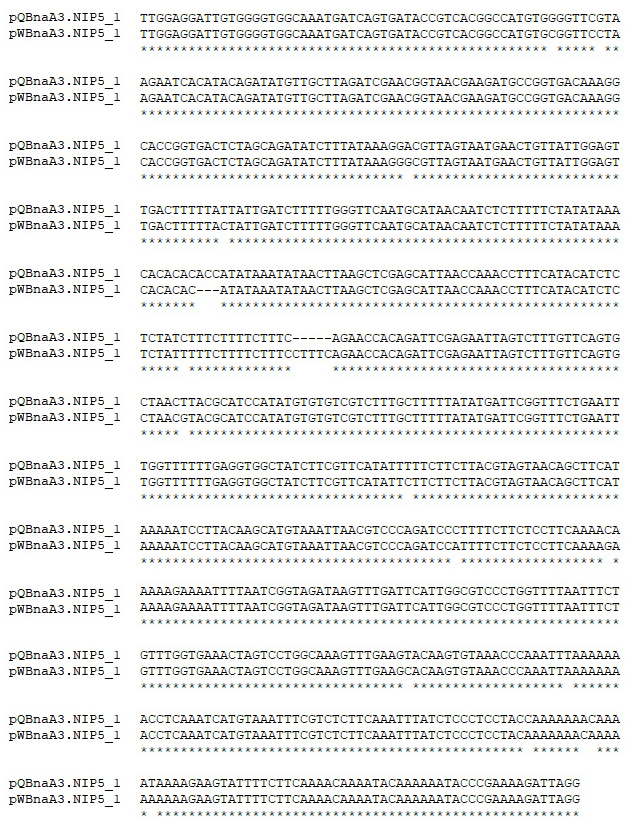

Supplement: S6 Fig — Red boxes indicate different TEs in BnaA3.NIP5;1Q promoter and BnaA3.NIP5;1W promoter. Green line indicates target repeat sequence and blue line indicates invert terminal sequence. (DOCX) [file pgen.1009661.s006.docx]

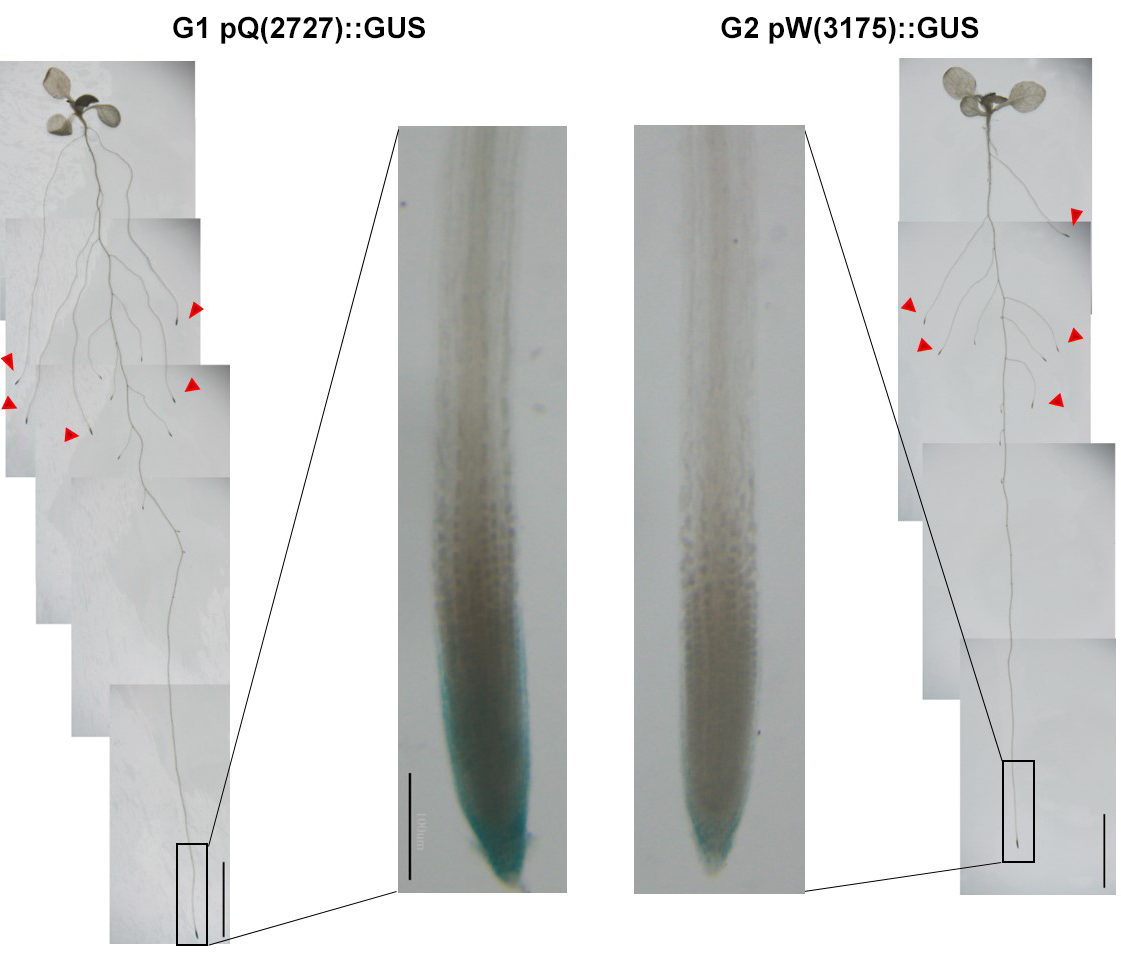

Supplement: S7 Fig — Seedlings of transgenic plants were grown on solid medium containing 0.3 μM B for 10 d. GUS activity are indicated by red triangle. Scar bar, 5 mm in the whole plant image and 100 μm in root tip image. (TIF) [file pgen.1009661.s007.tif]

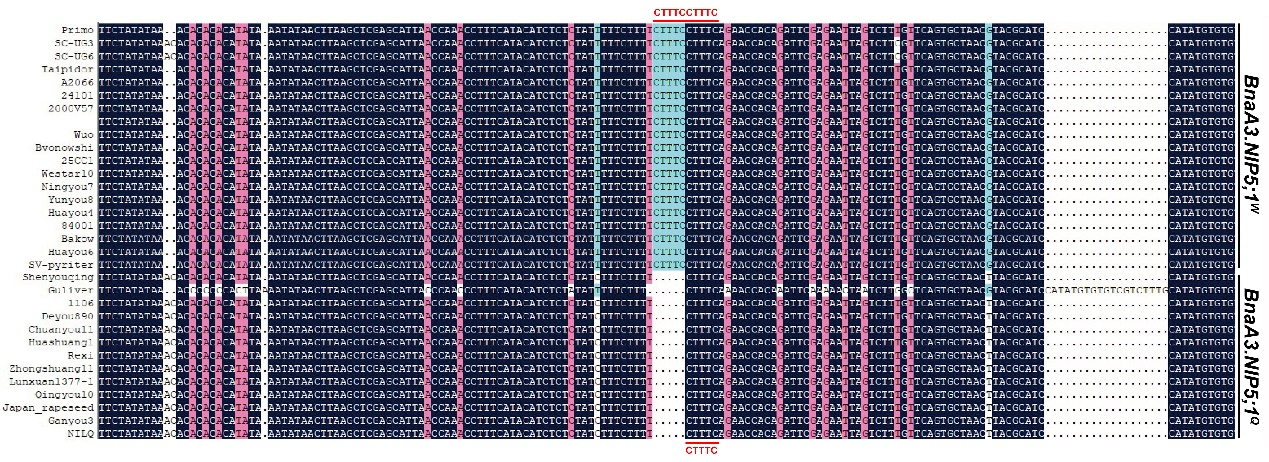

Supplement: S8 Fig — All varieties can be divided into two haplotype BnaA3.NIP5;1Q and BnaA3.NIP5;1W based on one or two CTTTC copies. Red bar indicated CTTTC repeats. (TIF) [file pgen.1009661.s008.tif]

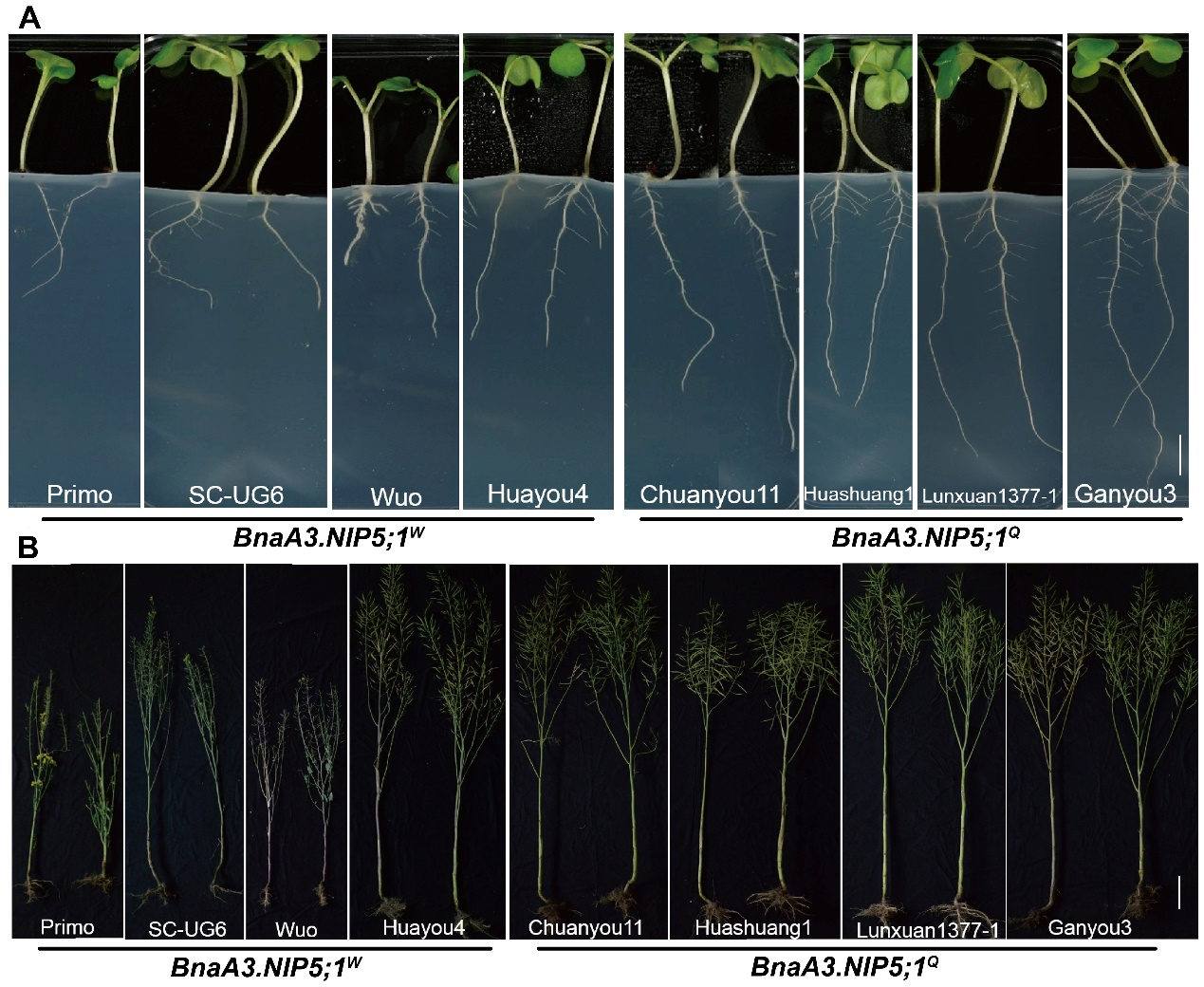

Supplement: S9 Fig — (A) Root growth appearance of the representative varieties under low B (0.1 μM) condition for 10 d. Seedlings of representative varieties were grown on the solid medium containing 0.1 B for 5 d. Scale bar, 1 cm. (B) Phenotype of the representative varieties grown at B-deficient field plot. Scale bar, 20 cm. (TIF) [file pgen.1009661.s009.tif]

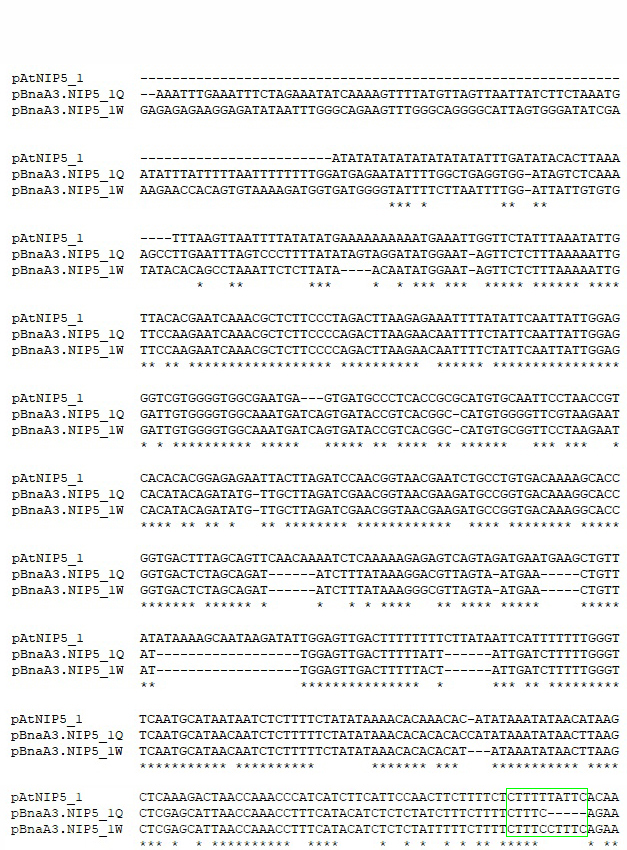

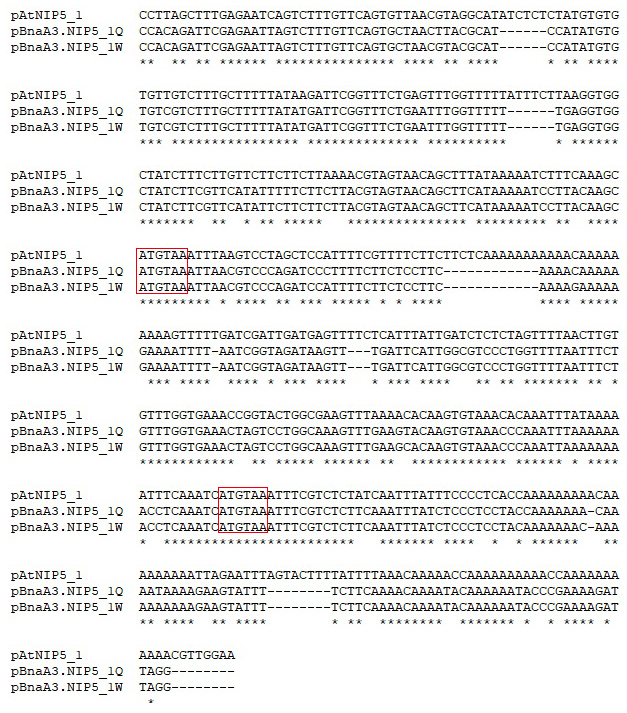

Supplement: S10 Fig — Green box indicate the CTTTC repeats and Red boxes indicate the uORF in the 5’UTR. (DOCX) [file pgen.1009661.s010.docx]

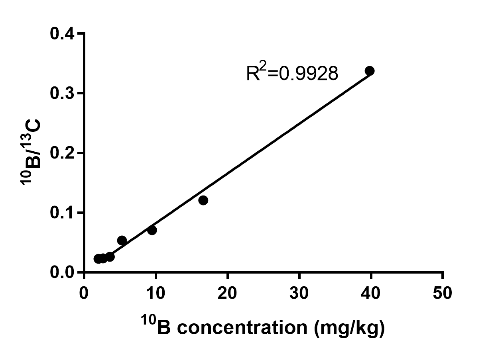

Supplement: S11 Fig — Rapeseed seedlings were grown on 0 B solid medium for 10 d, then collected and ground into powder after dried. 0, 20, 40, 100, 200, 400, 1000 μl 10 mg/L 10B were added, respectively, and mixed with the prepared powder, after absorption for 48 h, re-dried and ground to powder. After digestion, 10B concentration was determined as described above. And then 50 mg powder was pressed under 8 atm to make standard reference material. The standard reference material was put into the laser ablation chamber and scanned together with the root tips. (TIF) [file pgen.1009661.s011.tif]
