# Supplementary material for: Genetic variation of BnaA3.NIP5;1 expressing in the lateral root cap contributes to boron deficiency tolerance in Brassica napus
Source: PLoS Genet. 2021 Jul 1;17(7):e1009661. doi: 10.1371/journal.pgen.1009661 (PMC8279314; doi:10.1371/journal.pgen.1009661)
Supplement: S1 Table — (DOCX) [file pgen.1009661.s012.docx]

Table. List of the primers used in this stud.

| Primers for pQ::BnaA3.NIP5;1-GFP vector | | |
| --- | --- | --- |
| BnaA3.NIP5;1CDS-F | CGGAATTCATGTCTCCGCCGGAGGCT | |
| BnaA3.NIP5;1CDS-R | GCTCTAGAACGACGGAAGCTTCTAACCTGA | |
| Promoter-F | TTGGCGCGCCCCAATGTTTTGATTTTACTTTTGGG | |
| Promoter-R | GGAATTCCCTAATCTTTTCGGGTATTTTTTGTATT | |
|  | | |
| Primers for RNAi vector | | |
| RNAi-F | GGATCCGGCGCGCCTTTCAGAACCACAGATTCGAG | |
| RNAi-R | TCTAGACCATGGTTCAAACTTTGCCAGGACTAG | |
|  | | |
| Primers for the qRT-PCR analysis | | |
| qBnaA3.NIP5;1-F | TTGCCGGTGATGGGAAACCG | |
| qBnaA3.NIP5;1-R | CCTGCGCATGCCGCGTTG | |
| BnaEF1-α-F | GCCTGGTATGGTTGTGACCT | |
| BnaEF1-α-R | GAAGTTAGCAGCACCCTTGG | |
| BnaTubulin-F | CAGCAATACAGTGCCTTGAGTG | |
| BnaTubulin-R | CCTGTGTACCAATGAAGGAAAGCC | |
| AtActin-F | GTTCCAGCCCTCGTTTGTG | |
| AtActin-R | CAAGTGCTGTGATTTCTTTGCTC | |
| AtEF1α-F | TGGTGACGCTGGTATGGTTA | |
| AtEF1α-R | TCCTTCTTGTCCACGCTCTT | |
|  | | |
| Primers for *Xenpus laevis* oocyte expression vector | | |
| BnaA3.NIP5;1-GFP-F | | GAAGATCTATGTCTCCGCCGGAGGCT |
| BnaA3.NIP5;1-GFP-R | | GGACTAGTTTAGTGGTGGTGGTGGTGGTGT |
|  | | |
| Primers for the in-situ RT-PCR analysis of BnaA3.NIP5;1 | | |
| BnaA3.NIP5;1-F | TTGCCGGTGATGGGAAACCG | |
| BnaA3.NIP5;1-R | CCTGCGCATGCCGCGTTG | |
|  | | |
| Primers for pQ::BnaA3.NIP5;1-GUS vector | | |
| pQ::BnaA3.NIP5;1-F | ATGCCTGCAGGGGCGCGCCCCAATGTTTTGATTTTAC | |
| pQ::BnaA3.NIP5;1-R | TCCCCCGGGAAACGACGGAAGCTTCTAACCTGACG | |
|  | | |
| Primers for GUS activity analysis | | |
| G1/2-F | TTGGCGCGCCCCAATGTTTTGATTTTACTTTTGGG | |
| G3-F | TTGGCGCGCCACTAGAGATTTTTTCCGCGCTT | |
| G4-F | TTGGCGCGCCTTATAACAAGAGAGAATTTGTTGAATATACAT | |
| G5-F | TTGGCGCGCCATATGGAATAGTTCTCTTTAAAAATTGTTC | |
| G6-F | TTGGCGCGCCTTATAACAATATGGAATAGTTCTCTTTAAAAAT | |
| G-R | GGAATTCCCTAATCTTTTCGGGTATTTTTTGTATT | |
| G7/8-F | TTTCTTTCCTTTCAGAACCAC | |
| G7/8-R | CTGAAAGGAAAGAAAAGAAAGATAGAGAGATGTATGAAAGG | |
| G9/10-F | TTTCTTTCAGAACCACAGATTC | |
| G9/10-R | TGGTTCTGAAAGAAAAGAAAAATAGAGAGATGTATGAAAGG | |
|  | | |
| Primers for the resequencing of -849 to -1 of BnaA3.NIP5;1 promoter | | |
| 849-F | ATATGGAATAGTTCTCTTTAAAAATTGTTC | |
| 849-R | AATACAAAAAATACCCGAAAAGATTAG | |
|  | | |
| Primers for the genotyping of NILs | | |
| N-F | GCGACTACTTGTTCGTGTCGTG | |
| N-R | GGTCATTTGGTAAAAGATAATGAG | |
