# Supplementary material for: Genetic variation of BnaA3.NIP5;1 expressing in the lateral root cap contributes to boron deficiency tolerance in Brassica napus
Source: PLoS Genet. 2021 Jul 1;17(7):e1009661. doi: 10.1371/journal.pgen.1009661 (PMC8279314; doi:10.1371/journal.pgen.1009661)
Supplement: Attachment — Submitted filename: Response to reviewers.docx [file pgen.1009661.s013.docx]

**Response to the comments of two Reviewers**

Reviewer #1: In the manuscript entitled “Genetic variation of BnaA3.NIP5;1 expressing in the lateral root cap contributes to boron deficiency tolerance in Brassica napus”, the authors investigated the natural genetic variation in BnaA3.NIP5; 1 gene, which they identified but has not functionally characterized as a key determinant of low-B tolerance in B. napus in their previous studies. It was found that that a CTTTC tandem repeat in the 5’UTR of BnaA3.NIP5;1 altered its expression level, and associated with plant growth and seed yield. Though the orthologue of this gene in Arabidopsis has been well characterized, novel data and strong evidence were provided in this study to tell a novel story on the contribution of a superior allele to low-B tolerance of B. napus that requires high B supplies. Overall, the results are clear and the manuscript is easy to follow. Here I list my concerns for suggestion.

1 The CTTTC tandem repeat in the 5’UTR underlies the difference in expression levels in parental lines, NILs and natural populations. Then how does the CTTTC tandem repeat works? Does this repeat hinder the gene translation? Typically, the sequence variation in UTRs play roles on mRNA stability and translation, but not on transcription. More data should be provided to make the story clear.
Response: Thank you for your suggestion. There were two uORFs in the 5’UTR of *AtNIP5;1*, which controlling the *AtNIP5;1* mRNA degradation under high B conditions. However, there no difference in these two uORFs between *BnaA3.NIP5;1* and *AtNIP5;1*. In this research, we found that CTTTC tandem repeat alters the expression levels, similar results were found in other researches (Si et al., Nat Genet 2016, A tandem repeat of the CCATTC sequence in the 5′ UTR alters OsSPL13 expression), but the regulation mechanism still unknown and more studies need to be done in future. According to your suggestion, we add more information in Line 290-294 in Discussion section of the revised manuscript.
“However, the activities of pQ::BnaA3.NIP5;1-GFP and pW::BnaA3.NIP5;1-GFP differed when they were expressed in *nip5;1-1* (Fig. 4), it is possible that *Brassica napus* and *Arabidopsis* show the same trans-factor or transcript factor to regulate the CTTTC element, and further study can be carried out in *Arabidopsis* to found the upstream regelation element of *BnaA3.NIP5;1*.”

Reference: Si, L., Chen, J., Huang, X. et al. OsSPL13 controls grain size in cultivated rice. Nat Genet 48, 447–456 (2016).

2 Besides the phenotypic data on primary root length and productivity, the data on B uptake and/or accumulation are necessary to solidify the allelic difference in natural population (line 215-235) .
Response: We appreciate you raising this point. Indeed, BnaA3.NIP5;1 functions as a boric acid channel. However, due to the specifically expression pattern, BnaA3.NIP5;1 facilitates B uptake into root meristem but not whole root. We also found that QY10 (B-efficient cultivar) had a higher B concentration than W10 (B-inefficient cultivar) only in the root meristem region. When we measured the whole root or plant B concentration, we found that W10 had a higher B concentration than W10. This is the reason that we didn’t measure the data on B uptake or concentration in natural population and it is difficult for us to measure the root meristem B concentration using LA-ICP-MS.

3 some typos:
L58 Under low-B conditions, B can be taken up plants via two different mechanisms.
Response: We appreciate you raising this point. We rewrite this sentence in the revised manuscript.
“Under low-B conditions, B can be taken up via two different mechanisms in plants.”

L59 “Channel-mediated facilitated transport via NOD26-LIKE MAJOR INTRINSIC PROTEIN5;1 (NIP5;1), which encodes a channel protein belonging to the aquaporin family; this protein has been shown to facilitate B uptake from the soil in Arabidopsis roots.
Response: We appreciate you raising this point. We rewrite this sentence in the revised manuscript.
“Channel-mediated facilitated transport via NOD26-LIKE MAJOR INTRINSIC PROTEIN5;1 (NIP5;1) encodes a channel protein belonging to the aquaporin family.”

Reviewer #2: Boron is essential for plant development and reproduction. In this study, the authors characterized the molecular mechanism of BnaA3.NIP5;1 in boron deficiency response. They found that higher expression of BnaA3.NIP5;1 in B. napus varieties conferred low-boron tolerance. Further analysis revealed that a CTTTC tandem repeat in the 5’UTR of BnaA3.NIP5;1 determined its expression level. They also convincingly demonstrated that BnaA3.NIP5;1 transport boron into root tips and promote root growth under B-deficiency condition. Overall, this study provides new insights into the low-B tolerance and a novel perspective on the genetic improvement of boron efficiency in B. napus.

However, there are still a few issues need to be addressed.

1. The authors claimed that B taken up by BnaA3.NIP5;1 was used only for local root growth, however changing the expression of BnaA3.NIP5;1 dramatically influence the shoot growth and yield. Therefore, the tissue specific expression of BnaA3.NIP5;1 should be present. Is there any possibility that the BnaA3.NIP5;1 also has function for boron transport at SAM cells, which contribute to the shoot growth. If BnaA3.NIP5;1 only expressed in lateral root cap and affected root tip boron uptake and distribution, the authors should propose a possible mechanism to explain the impact of BnaA3.NIP5;1 for shoot growth.
Response: Thank you for your suggestion. Indeed, we analyzed the *BnaA3.NIP5;1* expression pattern in QY10 and W10 in our previous research (Hua et al., Plant Cell Environ 2016), results show that *BnaA3.NIP5;1* only expressed in the root. To explain the impact of *BnaA3.NIP5;1* for shoot growth, we added more information in Line 265-266 in Discussion section of the revised manuscript.

“With the well-developed root, other boron related transporters uptake more B into roots and jointly promote shoot growth with *BnaA3.NIP5;1*.”

Reference: Hua, Y.P. et al. Transcriptomics-assisted quantitative trait locus fine mapping for the rapid identification of a nodulin 26-like intrinsic protein gene regulating boron efficiency in allotetraploid rapeseed. Plant Cell Environ. 39, 1601–1618 (2016).
2. In fig3E, it's hard to distinguish the QC from other regions in root tips, it seems that high content of boron was accumulated in root meristem region.
Response: We appreciate you raising this point. We used root meristem region instead of QC in the revised manuscript.

3. Given the presence of other boron related transporters in plant, how could the higher expression of BnaA3.NIP5;1 in lateral root cap to maintain higher boron distribution in root tip under boron deficient condition and promote root growth? Possible explanation should be discussed.
Response: Thank you for your suggestion. We added more information in Line 258-263 in Discussion section of the revised manuscript.
“However, transgenic *nip5;1-1* still had a growth defect, which indicates *NIP5;1* expressing in the differential region is also important for plant normal growth under B limitation. It was reported that AtBOR2 also had a higher expression level in the LRC cells and with a proximal polar localization (10). This suggests that BnaA3.NIP5;1 may cooperate with other B transporters to maintain high B concentration in the root meristem region under B-limitation.”

Reviewer #3: Boron (B) is an essential element for plant growth and development. B nutrition is of great important for productivity of crops like rapeseed. In this manuscript He and her/his coworkers characterized a previously identified B transporter gene NIP5;1 for B-dependent root growth. They further discovered a CTTTC tandem repeat in the 5’-UTR of NIP5;1 that was capable to regulate the gene expression levels, and the corresponding natural variations were associated with root growth and seed yield under limited B conditions. This story highlights a novel function of B transporters in root development, and provides the favorite gene alleles for breeding low-B tolerant crops. The manuscript is well written with a proper language. Some minor points are needed to be considered for improving the quality of manuscript.


Minor points:
1) Line 161-162: this sentence can be “ By contrast, the shoots development did not differ among all genotypes”.
Response: Thank you for your suggestion. We changed this sentence in the revised manuscript as “By contrast, there was no significant difference in shoot growth among all these genotypes”.

2) Line 172-173: this sentence can be moved to the materials and methods part.
Response: Thank you for your suggestion. We moved this sentence to the materials and methods part in the revised manuscript.

3) Line 285-285: As shown in Figure 4, the activities of pQ and pW differed when they were expressed in *Arabidopsis*. Thus, this type of regulation may also exist in *Arabidopsis* even through the absence of CTTTC element in AtNIP5;1.
Response: We appreciate you raising this point. Based on our results, it is reasonable that this type of regulation may also exist for other genes in *Arabidopsis*.

4) Line 287-291: few words on the further research can be added.
Response: Thank you for your suggestion. We added more information in Line 290-294 in Discussion section of the revised manuscript.
“However, the activities of pQ::BnaA3.NIP5;1-GFP and pW::BnaA3.NIP5;1-GFP differed when they were expressed in *nip5;1-1* (Fig. 4), it is possible that *Brassica napus* and *Arabidopsis* show the same trans-factor or transcript factor to regulate the CTTTC element, and further study can be carried out in *Arabidopsis* to found the upstream regelation element of *BnaA3.NIP5;1*.”

Reviewer #4: Boron (B) is an essential micronutrient. But different plant species have different demand for B, among which allotetraploid rapeseed has a special high B demand. Deficiency in B supply usually caused serious yield loss in rapeseed. However, there is also a very large genetic difference in rapeseed resistance to B deficiency, which lies the base of the current study by He et al. This is a followed study from the same lab. In their previous studies, they identified a major QTL qBED-A3a and the possible candidate gene BnaA3.NIP5;1, whose expression is higher in B efficient variety QY10, but there is no difference in amino acid sequence with that in the B inefficient variety W10. In this study, the authors tried to elucidate why BnaA3.NIP5;1 expression in QY10 is higher, which also contributes to its better growth in B deficient soil. They found that BnaA3.NIP5;1 is polarly localized in the distal plasma membrane of LRC cells to facilitate B uptake into root tips, which also clarify why QC region has a higher B concentration. The interesting finding is that A CTTTC copy deletion within the 5’UTR increased BnaA3.NIP5;1 expression levels in QY10, whereas addition of the CTTTC into QY10 decreased the expression, so this tandem repeat is closely related with plant growth and seed yield. Finally, the authors presented solid evidence in the field test with natural populations and near-isogenic lines that the varieties carrying BnaA3.NIP5;1^Q^ allete produced higher seed yield under low-B condition. Overall speaking, this is a excellent work to provide novel insight into low-B tolerance in rapeseed, and more important is that the elite allele of BnaA3.NIP5;1 could be used as a direct target for breeding low-B tolerant cultivars in future practice. All the experiments were well designed and the contents were presented logically and easy to follow.
minor suggestions:


1. Line 157-158: it is better to rephase the sentence into : we generated transgenic plants expressing pQ::BnaA3.NIP5;1-GFP or pW::BnaA3.NIP5;1-GFP in the background of *Arabidopsis* mutant nip5;1-1.
Response: Thank you for your suggestion. We rewrite this sentence in the revised manuscript.

2. It is interesting that B taken up by LRC sells is used only for local root growth. Is there any effect of these transformation on the seed production of the transgenic *Arabidopsis* plants?
Response: We appreciate you raising this point. When we expressed *BnaA3.NIP5;1* in *Arabidopsis* mutant *nip5;1*, which promoted root growth under B-limitation. However, transgenic *Arabidopsis* plants still had a growth defect, so that we did not investigate the effect of these transformation on the seed production of the transgenic *Arabidopsis* plants. These results also indicate that NIP5;1 expressed in the differential region, is also crucial for plant growth under B-limitation.

3. In the discussion, is it possible to give proposes how the tandem repeat of CTTTC functioning in regulating the expression of BnaA3.NIP5;1?
Response: Thank you for your suggestion. We added more information in Line 290-294 in Discussion section of the revised manuscript.
“However, the activities of pQ::BnaA3.NIP5;1-GFP and pW::BnaA3.NIP5;1-GFP differed when they were expressed in *nip5;1-1* (Fig. 4), it is possible that *Brassica napus* and *Arabidopsis* show the same trans-factor or transcript factor to regulate the CTTTC element, and further study can be carried out in *Arabidopsis* to found the upstream regelation element of *BnaA3.NIP5;1*.”
